# Association between abusive supervision and nurses’ withholding voice about patient safety: the roles of impression management motivation and speak up-related climate

**DOI:** 10.1186/s12912-024-01921-0

**Published:** 2024-04-22

**Authors:** Zhi-Ying Li, Yu-Pei Yang, Qian Wang, Mei-Xian Zhang, Cheng-Wen Luo, Ling-Feng Zhu, Tao-Hsin Tung, Hai-Xiao Chen

**Affiliations:** 1 Taizhou Hospital of Zhejiang Province affiliated to Wenzhou Medical University, Linhai, Zhejiang 317000 China; 2 Department of Hematology, Taizhou Hospital of Zhejiang Province affiliated to Wenzhou Medical University, Linhai, Zhejiang 317000 China; 3 Evidence-based Medicine Center, Taizhou Hospital of Zhejiang Province affiliated to Wenzhou Medical University, Linhai, 317000 Zhejiang China; 4 Taizhou Hospital of Zhejiang Province, Zhejiang University, Linhai, Zhejiang China

**Keywords:** Abuse, Motivation, Nurses, Patient safety, Voice, Climate, Speaking

## Abstract

**Background:**

Abusive supervision by the nurse manager significantly influences nurses’ withholding voice about patient safety. The role of impression management motivation and speak up-related climate is crucial in understanding their connection. This study aimed to explore the relationship between abusive supervision, impression management motivation, speak up-related climate, and withholding voice about patient safety.

**Methods:**

This cross-sectional study employed a convenience sampling method to recruit 419 clinical nurses from Taizhou Hospital, Zhejiang Province, China, between 1 November 2022 and 31 January 2023. The study adhered to the STROBE checklist. Abusive supervision and impression management motivation were assessed using the Chinese versions of the Abusive Supervision Scale and the Impression Management Motivation Scale, respectively. Withholding voice about patient safety and speak up-related climate were identified using the Chinese version of the Speaking Up about Patient Safety Questionnaire.

**Results:**

Nurse leaders’ abusive supervision (β=0.40, *p*<0.01) and nurses’ impression management motivation (β=0.10, *p*<0.01) significantly and positively influenced nurses’ withholding voice about patient safety. We introduced impression management motivation as a mediating variable, and the effect of abusive supervision on nurses’ withholding voice decreased (β from 0.40 to 0.38, *p*< 0.01). Nurses’ speak up-related climate played a moderating role between abusive supervision and impression management motivation (β= 0.24, *p*<0.05).

**Conclusions:**

Abusive supervision by nursing leaders can result in nurses withholding voice about patient safety out of self-protective impression management motives. This phenomenon inhibits nurses’ subjective initiative and undermines their proactive involvement in improving patient safety, and hinders the cultivation of a culture encouraging full participation in patient safety, which should warrant significant attention.

## Background

Speaking up about patient safety not only gained substantial attention as a fundamental strategy for enhancing service quality and ensuring patient safety [1], but also has the potential to avert adverse events, enhance team performance, and cultivate a conducive learning climate [2, 3]. Withholding voice is an intentional act of withholding ideas, information, and opinions that improve patient safety from verbal expression [1]. From the clinical viewpoint, nurses frequently exhibit hesitancy in speaking up about patient safety, and ultimately choose to withhold their voices [4].

Leaders play a crucial role in shaping the behaviours of their subordinates [5]. Effective nursing leadership can positively impact both the work environment and patient safety [6–10]. Scholars have increasingly recognized the importance of ineffective leadership behaviours such as abusive supervision in influencing subordinates and organisations [11]. Abusive supervision refers to subordinates’ perceptions of the extent to which supervisors engage in the persistent display of hostile verbal and non-verbal behaviours [12]. Nurse leaders have heavy workloads, higher risks, and more time constraints. This makes it easier for them to enforce abusive supervision [13]. Multiple adverse outcomes triggered by abusive supervision in healthcare have been reported, including increased intent to resign, and reduced psychological empowerment [14]. The withholding voice by subordinates was a direct consequence of experiencing abusive supervision [15].

**Fig. 1:**
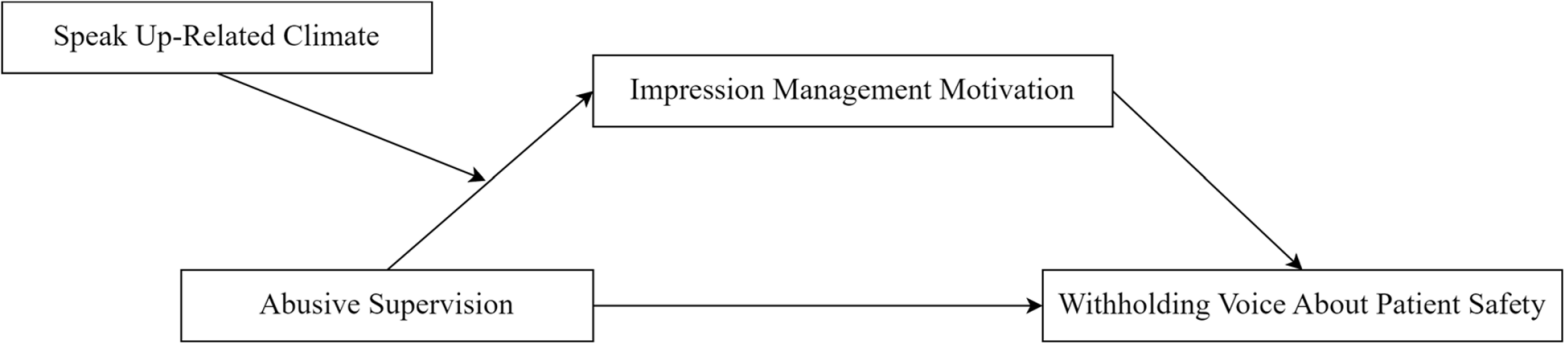
Abusive supervision and withholding voice about patient safety: A hypothetical theoretical model of the role of impression management motivation and speak up-related climate.

Impression management motivation refers to ‘the extent to which individuals are motivated to control the perception others have of them’ [16]. This motivation is contingent on the context, and individuals who display motivation for impression management are influenced by factors such as leadership styles and the external climates [17, 18]. Previous studies in China have demonstrated that the impression management motivation could serve as a mediating factor between leadership behaviour and the voice behaviour [19, 20]. In addition to abusive supervision as a leadership behaviour, organisational climate also affects impression management motivation, and consequently the withholding voice about patient safety [12, 20, 21]. Speak up-related climates cover various aspects that are relevant for withholding voice, including psychological safety, leadership, and an encouraging environment [1]. Therefore, we constructed the theoretical model shown in Fig. 1.

This study aimed to examine the association between abusive supervision and nurses’ withholding voice about patient safety, and further explored how nurses’ impression management motivation and speak up-related climate influenced this relationship. We proposed the following hypotheses:

- Hypothesis 1 (H1): Abusive supervision is positively associated with nurses’ withholding voice about patient safety.
- Hypothesis 2 (H2): Impression management motivation mediates the relationship between abusive supervision and nurses’ withholding voice about patient safety.
- Hypothesis 3 (H3): Speak up-related climates positively moderate the association between abusive supervision and impression management motivation.

## Methods

### Design

This was a hospital-based cross-sectional study and adhered to the guidelines provided by the Strengthening the Reporting of Observational Studies in Epidemiology (STROBE) checklist (Appendix S1).

### Setting and sample

This study utilized a convenience sampling method to survey clinical nurses from Taizhou Hospital, Zhejiang Province, China. The choice of this method was influenced by time and resource constraints [22]. Taizhou Hospital has been dedicated to providing a wide range of clinical acute treatments to the residents of Taizhou and its environs. The hospital offers educational and clinical training opportunities to medical and nursing students, and serves as a venue for various scientific projects [5].

At present, the hospital employs a total of 1,413 clinical nurses, of which 1,356 are females and 57 are males. A manual field survey was conducted in the wards over three months, from 1 November 2022 to 31 January 2023. A pilot survey was first conducted with small samples (*n*=10) to test the reliability of the scale before carrying out a formal survey. The preliminary results revealed that the Cronbach’s α for the Abusive Supervision Scale, Impression Management Motivation Scale, the Speak Up-Related Climate Scale, and the Speaking Up about Patient Safety Behaviours Scale were 0.93, 0.91, 0.84, and 0.85, respectively. Subsequently, after considering the scale’s structural validity, question 4 was excluded from the Speak Up-Related Climate Scale.

The sample size was calculated using the G*Power program (version 3.1). The study employed a linear multiple regression approach with an effect size of 0.05 [23]. We established an α level of 0.05 to control the acceptable Type I error rate. To minimize Type II errors, a desired statistical power (1-β error probability) of 0.9 was targeted. The study included 12 variables, comprising eight sociodemographic characteristics and four dimension-associated scales. The calculated minimum sample size required was 390 nurses. Considering the potential invalidity rate of questionnaires, we determined that 427 nurses were necessary for participation in this study. Ultimately, the study involved 427 nurses from a total population of 1413 nurses, and all 427 distributed questionnaires were successfully collected. After removing eight questionnaires with abnormal responses, 419 questionnaires remained valid, resulting in an effective response rate of 98.13% [22].

### Study instruments

Demographics included sex, age, marital status, education, professional categories, job tenure (years), monthly income (RMB), and department.

Abusive supervision was assessed using 15 items derived from Tepper’s scale [12], and translated and revised by Sun et al. [24]. Participants rated these items on a five-point Likert scale, ranging from one (strongly disagree) to five (strongly agree). Sample items, such as ‘Makes negative comments about me to others’, were included in the assessment. The total score (ranging from 15 to 75) of the scale was the sum of the responses of each item, where higher scores indicated more abusive supervision by the nurse manager. The scale exhibited excellent internal consistency, indicated by a Cronbach’s alpha coefficient of 0.95.

We used a survey instrument developed by Swiss scholars Richard et al. [25] and translated and revised by Yang et al. [26], to assess a nurse’s speaking up-related behaviours and speak up-related climate. To evaluate speak up-related behaviours, this scale employed 11 items organized into three subscales: perceived concerns (α=0.73; three items), speaking up (α=0.79; four items), and withholding voice (α=0.86; four items) [25]. These scales utilise a five-point Likert scale, ranging from ‘never’ (0 times) to ‘very often’ (more than ten times), and are based on the timeframe of ‘in the past month’. The total score (ranging from 11 to 55) of the scale was the sum of the responses of each item. Higher total scores indicate more frequent occurrences of speaking up and withholding voices.

To evaluate speak up-related climate, this scale (α=0.70) employed 11 items organized into three subscales: psychological safety for speaking up (α=0.72; five items), encouraging environment for speaking up (α=0.89; three items), and resignation (α=0.79; three items) [25]. Respondents rated their answers on a seven-point Likert scale, ranging from ‘strongly disagree with this statement’ to ‘strongly agree with this statement’. The total score (ranging from 11 to 77) of the scale was the sum of the responses of each item. Consequently, a higher total score indicates an increased level of psychological safety and a more encouraging environment for speaking up in the workplace.

Impression management motivation was assessed utilizing a self-report ten-item scale created by Rioux and Penner [27], and translated and revised by Wang et al. [28]. Participants rated these items on a five-point Likert scale, ranging from one (strongly disagree) to five (strongly agree). The total score (ranging from 10 to 50) of the scale was the sum of the responses of each item, where higher scores indicated more impression management motivation. Statements like ‘To avoid looking bad in front of others’ were included in the scale. A Cronbach’s alpha value of 0.94 was obtained, which indicates outstanding reliability of the scale. All English questionnaires are shown Appendix S2.

### Ethics considerations

This study received approval from the Ethics Committee of Taizhou Hospital, Zhejiang Province (approval number: K20220850), in compliance with the guidelines of the Institutional Ethics Committee and the principles outlined in the Declaration of Helsinki. Informed consent was obtained from all participants. Confidentiality of all participants’ information was strictly maintained, and each participant had the right to withdraw from the study at any time.

### Data analysis

The study employed several statistical methods, including descriptive statistical analysis to present nurses’ demographic information, t-tests (or ANOVA), and Pearson correlation analysis to investigate the correlations between abusive supervision, impression management motivation, speak up-related climate, and withholding voice about patient safety. The correlation coefficient is interpreted with <0.3 as weak and >0.7 as strong [29]. We selected variables that were P < 0.2 in univariate analyses and clinically relevant variables were included as control variables in the next step of the multiple linear regression analyses [30]. A hierarchical linear regression analysis was conducted to examine the associations and the mediating as well as moderating effects of these variables. The mediators were tested by computing bias-corrected 95% confidence intervals using bootstrapping with n = 5,000 re-samples employing the PROCESS procedure in SPSS [31]. The analyses were conducted using SPSS 21.0 software.

## Results

### Demographic information

As shown in Table 1, 50.60% of the nurses were aged 30 years and above among the 419 nurses who participated in the survey. Female nurses made up 98.57% of the sample. A majority of the nurses (71.36%), possessed a bachelor’s degree or higher. 32.70% of the surveyed nurses reported a monthly income exceeding RMB 10,000. Approximately 38.90% were nurse practitioners, 29.36% were nurse leaders with intermediate professional nursing titles and above. Ninety (21.47%) had been working for 0–1 years, 68 (16.23%) had been working for 2–4 years, 91 (21.72%) had been working for 5–9 years, 85 (20.29%) had been working for 10–14 years, and 85 (20.29%) had been working for more than 15 years. Moreover, 26.25% were from the Internal medicine department, 20.29% from the Surgery department, 19.57% from the Emergency department, and 33.89% from other departments.

**Table 1:** Demographic information and univariate analysis of factors associated with withholding voice (*n* = 419)

Characteristic	Category	*n*(%)	Withholding voice M±SD	t-test or ANOVA	*P*
Gender	Female	413(98.57**)**	1.67±0.63	1.78	0.08
	Male	6(1.43)	2.13±0.38		
Age (years)	21–24	107(25.53)	1.68±0.56	0.54	0.58
	25–29	100(23.87)	1.73±0.73		
	≥30	212(50.60)	1.65±0.61		
Marital status	Married	236(56.32)	1.65±0.64	0.74	0.46
	Unmarried or divorced	183(43.68)	1.70±0.61		
Education	Specialized training school	120(28.64)	1.70±0.60	0.63	0.53
	Undergraduate and above	299(71.36)	1.66±0.64		
Professional categories	Nurses	133(31.74)	1.72±0.65	2.08	0.13
	Nurse practitioners	163(38.90)	1.71±0.65		
	Nurse-in-charge or above	123(29.36)	1.58±0.57		
Job tenure (years)	0–1	90(21.47)	1.73±0.54	0.82	0.52
	2–4	68(16.23)	1.66±0.67		
	5–9	91(21.72)	1.70±0.69		
	10–14	85(20.29)	1.70±0.70		
	≥15	85(20.29)	1.57±0.54		
Monthly income (RMB)	≤10000	282(67.30)	1.71±0.64	1.59	0.11
	>10000	137(32.70)	1.60±0.60		
Department	Internal medicine	110(26.25)	1.60±0.58	0.57	0.68
	Surgery	85(20.29)	1.70±0.65		
	Emergency	82(19.57)	1.72±0.67		
	Orthopaedics	40(9.55)	1.73±0.49		
	Others	102(24.34)	1.67±0.67		

We further conducted t-test and ANOVA on categorical variables such as gender, age, professional categories, monthly income, and education to identify the factors that can influence withholding voice to carry out the next regression analysis. The results of the analyses are also shown in Table 1.

### Correlations between study variables

Table 2 presents the means, standard deviations, and Pearson correlation coefficients for all continuous variables. It is observed that the mean score of nurse managers’ abusive supervision was 1.31 ± 0.48, and the mean score of nurses withholding voice about patient safety was 1.67 ± 0.63. The mean score for nurses’ impression management motivation was 2.62 ± 0.93, while the speak up-related climate had a mean score of 5.56 ± 0.71. We observed a moderate and positive correlation between abusive supervision and nurses’ withholding voice about patient safety (r = 0.31, p<0.01). A very weak and positive correlation between abusive supervision and impression management motivation (r = 0.12, p<0.01). There was also a weak and negative correlation between abusive supervision and speak up-related climate (r = -0.21, p<0.01). Additionally, nurses’ withholding voice about patient safety exhibited a weak and positive correlation with impression management motivation (r = 0.20, p<0.01).

**Table 2:** Pearson correlation between dependent and independent variables (*n* = 419)

	M	SD	Abusive supervision	Impression management motivation	Speak up-related climate	Withholding voicex
Abusive supervision	1.31	0.48	1.00	– –	– –	– –
Impression management motivation	2.62	0.93	0.12*	1.00	– –	– –
Speak up-related climate	5.56	0.71	-0.21**	0.04	1.00	– –
Withholding voice	1.67	0.63	0.31**	0.20**	-0.05	1.00

^*^p <0.05 ; **p <0.01

### Multiple hierarchical linear regression models

Multiple linear regression analyses were conducted to test our hypotheses. Gender, age, professional categories, and monthly income that may influence nurses to withholding voice were selected as control variables, testing the associations between abusive supervision, impression management motivation, speak up-related climate, and nurses’ withholding voice. Additionally, impression management motivation was tested as a potential mediator in the relationship between abusive supervision and withholding voice.

Results reported in Table 3 show that abusive supervision is significantly and positively associated with withholding voice (β= 0.40, *p* <0.01) (Model 2), which support H1. Subsequently, we introduced impression management motivation as a mediating variable, and the effect of abusive supervision on nurses’ withholding voice decreased (β from 0.40 to 0.38, *p *< 0.01) (Model 2, Model 3). Furthermore, abusive supervision was found to be positively associated with impression management motivation (β= 0.23, *p* =0.02) (Model 5); and impression management motivation was positively associated with withholding voice (β= 0.10, *p* < 0.01) (Model 3). These findings suggest that impression management motivation partially mediates the relationship between abusive supervision and nurses’ withholding voice, thus offering support for H2. The regression analysis revealed a positive association between abusive supervision and withholding voice (β = 0.38, *p* < 0.01). The bootstrapped 95% confidence interval (LLCI: 0.00, ULCI: 0.05) did not contain zero, confirming the establishment of the mediating role of impression management motivation in the abusive supervision and nurses’ withholding voice relationship. Table 4 displays the results, which supports H1 and H2.

**Table 3:** Hierarchical multiple regression model for withholding voice and impression management motivation (n = 419)

	Withholding voice			Impression management motivation							
	Model 1	Model 2	Model 3	Model 4	Model 5		Model 6		Model 7		
Control variables											
Gender											
male vs female	0.44	0.26	0.24	0.25	0.14		0.12		0.07		
Age											
25–29 vs <25	0.15	0.12	0.14	-0.16	-0.18		-0.17		-0.17		
≥30 vs <25	0.20	0.20	0.22		-0.23	-0.23		-0.22		-0.22	
Professional categories											
Nurse practitioner vs Nurses	-0.13	-0.15	-0.17		0.20	0.18		0.16			0.16
Nurse-in-charge or above vs Nurses	-0.26	-0.28*	-0.29*		0.02	0.01		-0.01			-0.02
Monthly income (RMB) >10000 vs ≤10000	-0.07	-0.06	-0.04		-0.24*	-0.24*		-0.23*			-0.23*
Independent variable											
Abusive supervision		0.40**	0.38**			0.23*		0.26**			0.27**
Mediator											
Impression management motivation			0.10**								
Moderator											
Speak up-related climate								0.09			0.09
Abusive supervision× Speak up-related climate											0.24*
R^2^	0.02	0.12	0.14		0.04	0.05		0.06			0.06
ΔR^2^	0.02	0.09	0.02		0.04	0.01		0.00			0.01
F	1.63	7.69	8.15		2.61	3.12		2.96			3.08

^*^p <0.05 ; **p <0.01

**Table 4:** Mediating effect of impression management motivation (n = 419)

Effect	S.E.	t	*p*	LLCI	ULCI
0.40	0.06	6.56	<0.01	0.28	0.53
Direct effect of abusive supervision on withholding voice					
Effect	S.E.	t	p	LLCI	ULCI
0.38	0.06	6.21	<0.01	0.26	0.50
Indirect effect abusive supervision and withholding voice					
Effect	Boot S.E.			Boot LLCI	Boot ULCI
0.02	0.01			0.00	0.05

**Fig. 2:**
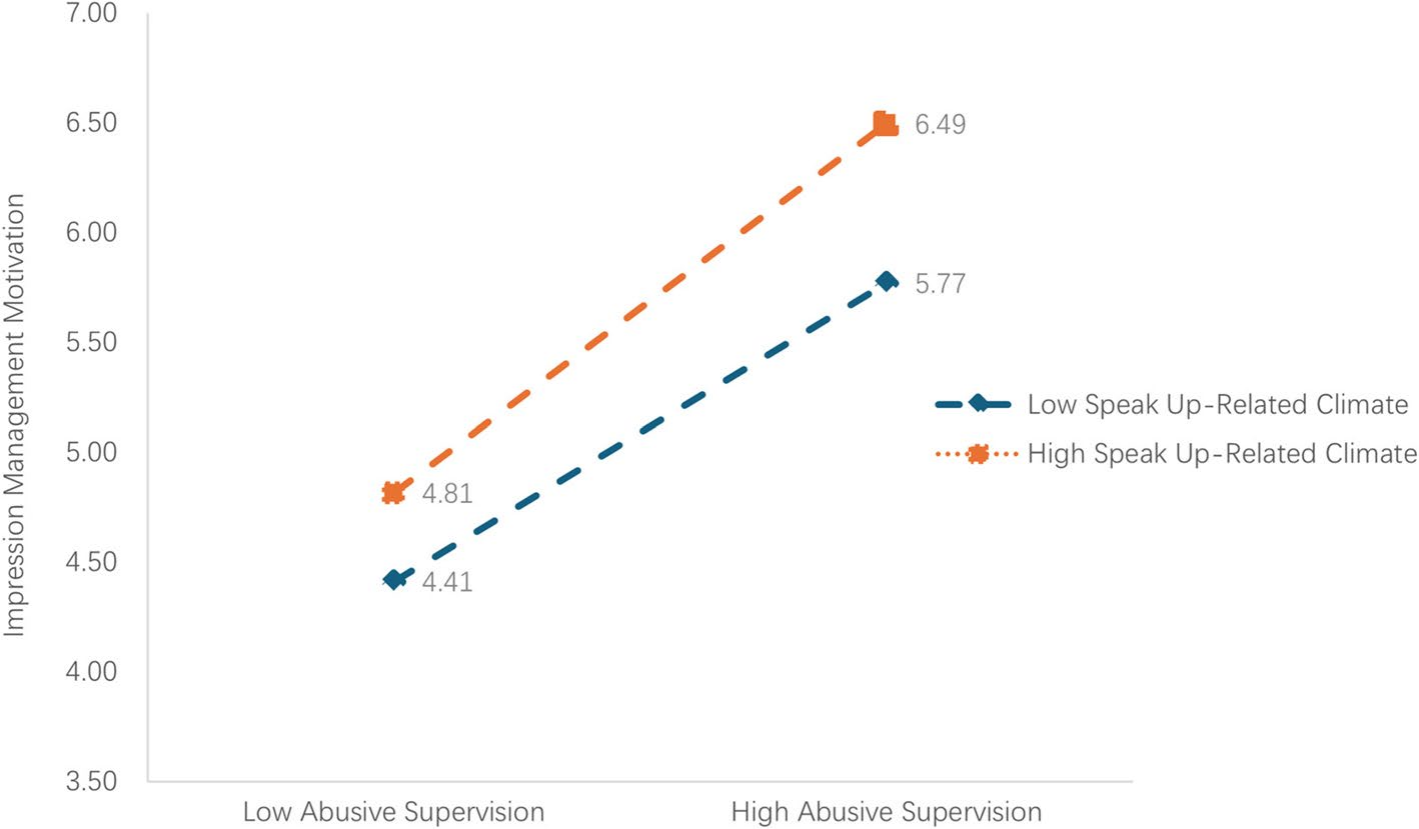
Moderated effect of speak up-related climate on the association between abusive supervision and impression management motivation.

According to Aiken and West’s recommendations, the data were centred by subtracting the mean value [32]. The findings revealed a positive association between the interaction term of speak up-related climate and abusive supervision and impression management motivation (β= 0.24, p<0.05) (Table 3, Model 7). This result indicates that speak up-related climate moderates the relationship between abusive supervision and impression management motivation, confirming H3. Figure 2 illustrates that the positive relationship between abusive supervision and impression management motivation is more pronounced in a high speak up-related climate.

## Discussion

This study showed that abusive supervision by nurse managers was significantly positively associated with nurses’ withholding voice about patient safety (Hypothesis 1), the influence of abusive supervision on nurses’ withholding voice about patient safety was partly mediated by impression management motivation (Hypothesis 2), and the positive relationship between abusive supervision and impression management motivation was moderated by the speak-up related climate (Hypothesis 3).

The scores for abusive supervision among nurse leaders in this study are similar to previous findings [33, 34]. This implies that abusive supervision is a low base rate phenomenon [35]. Our results further revealed relative lower impression management motivation scores than other academic studies [17, 18], and speak up-related climate scores were slightly higher than previous findings [36].This disparity may arise from variances in the diverse occupational backgrounds and geographical origins within the study population [17, 18, 37]. In addition, our results showed lower nurses’ withholding voice about patient safety scores than other academic studies [5, 38]. The reason for this might be that nurses’ withholding voice about patient safety could easily lead to the occurrence of medical hazards or adverse events, thus most nurses have lower frequencies of withholding voice [1]. However, there are still some nurses who choose to withhold their voices when encountering patient safety issues.

Consistent with other findings, this study indicated a positive correlation between abusive supervision by nurse managers and withholding voice about patient safety by nurses [38, 39]. When the nurse manager imposes abusive supervision on nurses, nurses are affected psychologically, and are concerned that they might be humiliated or punished by their nurse manager for speaking up, therefore, they choose to withholding voice [19, 38]. In addition, the abusive supervisor is mostly perceived by their subordinates as having authoritarian and despotic personalities, and speaking up about patient safety is perceived as questioning their management or challenging their authority, thus nurses choose to withholding voice about patient safety [2, 40, 41].

This study further found that impression management motivation played a partial mediating role in the relationship between abusive supervision and nurses’ withholding voice about patient safety. Previous researches have demonstrated that the impression management motivation could serve as a mediating factor between leadership behaviour and the voice behaviour [19, 20]. Nurses feel psychologically less secure and threatened when nurse managers engage in abusive behaviours such as angry tantrums, public criticisms, and inappropriately assigned blame against nurses [21, 42]. Individuals who feel threatened could be motivated to avoid damage to their personal image, hence, nurses may choose to withhold voice about patient safety at this point to avoid further abusive supervision by their leaders [21].

This study showed that speak up-related climate acted as a moderating factor between abusive supervision and impression management motivation, and nurses in a high speak up-related climate generated stronger impression management motivation. This organisational climate has often appeared as a moderating variable in previous academic studies [43–46]. Organisational climate is an intrinsic mechanism through which leadership behaviour influences subordinates’ motivation [1, 47]. At the individual leader level, abusive supervision influences impression management motivation. In addition, the organisational climate could also influence impression management motivation [21]. This implies that in a supportive speak up-related climate, nurses are more likely to exhibit impression management motivation and adopt impression management strategies.

This study explores a nursing management perspective for enhancing nurses’ willingness to speak up about patient safety. Nursing managers should increase their awareness of abusive supervision. Hospitals and other institutions should enhance leaders’ awareness of the hazards associated with abusive supervision, strengthen their self-control, and minimize the occurrence of abusive supervision [48]. Online and offline reporting centres could be established in hospitals to facilitate the reporting of abusive behaviours by nursing staff or patients [39].

### Limitations

There are several limitations in this study. First, based on the convenience sampling approach, the study population does not only introduce selection bias, but also the Hawthorne effect is inevitable [49]. Further studies could improve this limitation by random sampling methods. Second, due to only six male nurses in our study sample, results are affected greatly, which makes it very difficult to estimate gender disparities in withholding voice about patient safety. Third, there is a weak correlation observed among the key variables investigated in this study. Fourth, the study was carried out only in a single public hospital in the Taizhou region, potentially constraining the generalizability of the results. Therefore, future research could benefit from conducting surveys in multiple centres with larger samples to enhance the representativeness and generalizability of the results [22]. Finally, the study was cross-sectional and provided evidence rather than causal inferences. Thus, future research should consider longitudinal studies, such as cohort or case-control studies, to explore the dynamics and causal relationships between abusive supervision and nurses’ withholding voice about patient safety.

## Conclusion

In conclusion, nurse managers’ abusive supervision influences nurses’ withholding voice about patient safety. Impression management motivation partially mediated the relationship between abusive supervision and nurses’ withholding voice about patient safety. Speaker up-related safety climates moderated the relationship between abusive supervision and impression management motivation. The positive role of abusive supervision in promoting impression management motivation is enhanced in the presence of a more positive speaker up-related safety climate.

## Data Availability

The datasets used and/or analysed during the current study are available from the corresponding author on reasonable request.

## Appendix 1

STROBE Statement

**Table:**

	**Item No**	**Recommendation**	**Page No**
Title and abstract	1	(*a*) Indicate the study’s design with a commonly used term in the title or the abstract	2
		(*b*) Provide in the abstract an informative and balanced summary of what was done and what was found	2-3
**Introduction**			
Background/rationale	2	Explain the scientific background and rationale for the investigation being reported	4-5
Objectives	3	State specific objectives, including any prespecified hypotheses	5
**Methods**			
Study design	4	Present key elements of study design early in the paper	6
Setting	5	Describe the setting, locations, and relevant dates, including periods of recruitment, exposure, follow-up, and data collection	6-7
Participants	6	(*a*) Give the eligibility criteria, and the sources and methods of selection of participants	7
Variables	7	Clearly define all outcomes, exposures, predictors, potential confounders, and effect modifiers. Give diagnostic criteria, if applicable	7-9
Data sources/ measurement	8*	For each variable of interest, give sources of data and details of methods of assessment (measurement). Describe comparability of assessment methods if there is more than one group	7-9
Bias	9	Describe any efforts to address potential sources of bias	9-10
Study size	10	Explain how the study size was arrived at	7
Quantitative variables	11	Explain how quantitative variables were handled in the analyses. If applicable, describe which groupings were chosen and why	9-10
Statistical methods	12	(*a*) Describe all statistical methods, including those used to control for confounding	9-10
		(*b*) Describe any methods used to examine subgroups and interactions	N/A
		(*c*) Explain how missing data were addressed	7
		(*d*) If applicable, describe analytical methods taking account of sampling strategy	6
		(*e*) Describe any sensitivity analyses	N/A
**Results**			
Participants	13*	(a) Report numbers of individuals at each stage of study—eg numbers potentially eligible, examined for eligibility, confirmed eligible, included in the study, completing follow-up, and analysed	11
		(b) Give reasons for non-participation at each stage	N/A
		(c) Consider use of a flow diagram	N/A
Descriptive data	14*	(a) Give characteristics of study participants (eg demographic, clinical, social) and information on exposures and potential confounders	11-12; 29
		(b) Indicate number of participants with missing data for each variable of interest	N/A
Outcome data	15*	Report numbers of outcome events or summary measures	11-13
Main results	16	(*a*) Give unadjusted estimates and, if applicable, confounder-adjusted estimates and their precision (eg, 95% confidence interval). Make clear which confounders were adjusted for and why they were included	11-13
		(*b*) Report category boundaries when continuous variables were categorized	11;29
		(*c*) If relevant, consider translating estimates of relative risk into absolute risk for a meaningful time period	N/A
Other analyses	17	Report other analyses done—eg analyses of subgroups and interactions, and sensitivity analyses	N/A
**Discussion**			
Key results	18	Summarise key results with reference to study objectives	14-16
Limitations	19	Discuss limitations of the study, taking into account sources of potential bias or imprecision. Discuss both direction and magnitude of any potential bias	16-17
Interpretation	20	Give a cautious overall interpretation of results considering objectives, limitations, multiplicity of analyses, results from similar studies, and other relevant evidence	14-16
Generalisability	21	Discuss the generalisability (external validity) of the study results	16-17
**Other information**			
Funding	22	Give the source of funding and the role of the funders for the present study and, if applicable, for the original study on which the present article is based	19

*Give information separately for exposed and unexposed groups: not applicable

**Note**: An Explanation and Elaboration article discusses each checklist item and gives methodological background and published examples of transparent reporting. The STROBE checklist is best used in conjunction with this article (freely available on the Web sites of PLoS Medicine at http://www.plosmedicine.org/, Annals of Internal Medicine at http://www.annals.org/, and Epidemiology at http://www.epidem.com/). Information on the STROBE Initiative is available at www.strobe-statement.org.

## Appendix 2

### Questionnaires

#### Part I. Demographic information

1. Your Gender is: A. Female; B. Male
2. Your Age (years) is:
3. Your Marital status is: A. Married; B. Unmarried; C. Divorced
4. Your Education is:
  A Specialized training school; B. Undergraduate; C. Postgraduate and above
5. Your Professional categories is:
  A Nurses; B. Nurse practitioners; C. Nurse-in-charge; D. Associate Chief Nurse; E. Chief Nurse
6. Your Job tenure (years) is: A. 0–1; B. 2–4; C. 5–9; D. 10–14; E. ≥15
7. Your monthly income (RMB) is: A. ≤10000; B. >1000
8. Your department is:
  A Internal Medicine; B. Surgery; C. Emergency; D. Orthopedics; E. Others

## Abusive Supervision

### Part II. Completion of questionnaires

**Table:**

Abusive Supervision Questionnaire
1. Ridicules me
2. Tells me my thoughts or feelings are stupid
3. Gives me the silent treatment
4.Puts me down in front of others
5.Invades my privacy
6.Reminds me of my past mistakes and failures
7.Doesn't give me credit for jobs requiring a lot of effort
8.Blames me to save himself/herself embarrassment
9.Breaks promises he/she makes
10.Expresses anger at me when he/she is mad for another reason
11.Makes negative comments about me to others
12.Is rude to me
13.Does not allow me to interact with my coworkers
14.Tells me I'm incompetent
15. Lies to me

## Impression management motivation

**Table:**

Impression Management Motivation Questionnaire: What motivates you when you are actively working hard, providing help to your colleagues, or advising the hospital?
To avoid looking bad in front of others.
To avoid looking lazy.
To look better than my co-workers.
To avoid a reprimand from my boss.
Because I fear appearing irresponsible.
To look like I am busy.
To stay out of trouble.
Because rewards are important to me.
Because I want a raise.
To impress my co-workers

## Withholding voice and speak up-related climate

**Table:**

Survey items of the Speaking Up about Patient Safety Questionnaire
Over the last 4 weeks, how often…
Withholding voice
Did you choose not to bring up your specific concerns about patient safety?
Did you keep ideas for improving patient safety in your unit to yourself?
Did you remain silent when you had information that might have prevented a safety incident in your unit?
Did you not address a colleague (doctors and/or nurses) if he/she didn’t follow important patient safety rules, intentionally or unintentionally?
Speak Up-Related Climate
I can rely on my colleagues (doctors and/or nurses), whenever I encounter difficulties in my work.
I can rely on the shift supervisor (person in charge of a shift) whenever I encounter difficulties in my work.
The culture in my unit/clinical area makes it easy to speak up about patient safety concerns.
My colleagues (doctors and/or nurses) react appropriately, when I speak up about my concerns about patient safety.
In my unit/clinical area, I observe others speaking up about their patient safety concerns.
I am encouraged by my colleagues (doctors and/or nurses) to speak up about patient safety concerns.
I am encouraged by my shift supervisor (person in charge during a shift) to speak up about patient safety concerns.
When I have patient safety concerns it is difficult to bring them up
Having to remind staff of the same safety rules again and again is frustrating.
Sometimes I become discouraged because nothing changes after expressing my patient safety concerns.

## Abbreviations

STROBE: Strengthening the Reporting of Observational studies in Epidemiology
SPSS: Statistical product and service solutions
SD: Standard deviation
